# Spatial analysis of pneumococcal meningitis in São Paulo in the pre- and post-immunization era

**DOI:** 10.11606/S1518-8787.201905300118

**Published:** 2019-07-10

**Authors:** Danise Senna Oliveira, Francisco Chiaravalloti, Thiago Santos Mota, Daniel Brito de Araujo, Ana Marli Christovam Sartori

**Affiliations:** I Universidade Federal de Pelotas. Faculdade de Medicina. Departamento de Clínica Médica. Pelotas, RS, Brasil; II Universidade de São Paulo. Faculdade de Saúde Pública. Departamento de Epidemiologia. São Paulo, SP, Brasil; III Faculdade de Tecnologia de Botucatu. Departamento de Estatística. Botucatu, SP, Brasil; IV Universidade de São Paulo. Faculdade de Medicina. Departamento de Moléstias Infecciosas e Parasitárias. São Paulo, SP, Brasil

**Keywords:** Meningitis, Pneumococcal, prevention & control, Pneumococcal Vaccines, supply & distribution, Vaccination Coverage, Spatial Analysis, Geographic Information Systems, utilization

## Abstract

**OBJECTIVE:**

To analyze the pneumococcal meningitis incidence rates in the State of São Paulo, Brazil, by age group, municipalities and micro-regions, as well as the spatial distribution of pneumococcal meningitis incidence rates among children under 5 years old in the pre- (2005–2009) and post-vaccination (2011–2013) periods and its associations with socioeconomic variables and vaccination coverage.

**METHODS:**

The data source was the Brazilian Notifiable Diseases Information System. For the pre- and post-vaccination periods, thematic maps were built for pneumococcal meningitis incidence in under-5 children, by São Paulo state micro-regions, vaccination coverage and socioeconomic variables, using QGIS 2.6.1 software. Scan statistics performed by the SatScan 9.2 software were used to analyze spatial and spatiotemporal clusters in São Paulo municipalities and micro-regions. A Bayesian inference for latent Gaussian model with zero-inflated Poisson model through the integrated nested Laplace approximation was used in the spatial analysis to evaluate associations between pneumococcal meningitis incidence rates and socioeconomic variables of interest in São Paulo micro-regions.

**RESULTS:**

From 2005 to 2013, 3,963 pneumococcal meningitis cases were reported in São Paulo. Under-5 children were the most affected in the whole period. In the post-vaccination period, pneumococcal meningitis incidence rates decreased among this population, particularly among infants (from 4.17/100,000 in 2005 to 2.54/100,000 in 2013). Two clusters were found in pre-vaccination – one of low risk for pneumococcal meningitis, in the northwest of the state (OR = 0.45, p = 0.0003); and another of high risk in the southeast (OR = 1.62, p = 0.0000). In the post-vaccination period, only a high-risk cluster remained, in the southeast (RR = 1.97, p = 0.0570). In Bayesian analysis, wealth was the only variable positively associated to pneumococcal meningitis (RR = 1.026, 95%CI 1.002–1.052).

**CONCLUSIONS:**

Pneumococcal meningitis is probably underdiagnosed and underreported in São Paulo. Differentiated rates of pneumococcal meningitis diagnosis and reporting in each microregion, according to the São Paulo Index of Social Responsibility, might explain our results.

## INTRODUCTION

*Streptococcus pneumonia* e may cause serious life-threatening invasive diseases, such as pneumococcal meningitis (PM), sepsis and bacteremic pneumonia, as well as less serious but more common non-invasive diseases, such as non-bacteremic pneumonia and acute otitis media. Children aged ≤ 2 years, older adults, and persons of any age with chronic conditions are particularly affected^1^ .

Pneumococcal disease (PD) is a major cause of morbidity and mortality, particularly in developing countries. Crowded living conditions, early weaning, environmental pollution, smoking, presence of comorbidities and low socioeconomic status are risk factors for PD, especially when associated with each other and within age groups of greater risk, making regions with the lowest human development indexes (HDI) the ones with the highest pneumococcal disease incidence^2^ .

The introduction of pneumococcal conjugate vaccines into childhood immunization programs resulted in a substantial reduction of invasive pneumococcal disease (IPD) incidence rates in countries with high vaccination coverage^3^ . Universal childhood immunization with the 10-valent pneumococcal conjugate vaccine (PCV10) was introduced into the Brazilian National Immunization Program in 2010. The evaluation of the burden of pneumococcal disease, including PM, before and after the introduction of pneumococcal vaccine is important for assessing the impact of the vaccination program^4 , 5^ .

This study aimed to analyze the spatial distribution of PM incidence rates in under-5 children in the state of São Paulo (SP) before (2005–2009) and after (2011–2013) PCV10 introduction into universal childhood immunization. Associations between the PM distribution in SP administrative micro-regions and socioeconomic variables and vaccination coverage were analyzed through spatial statistical methods. Finally, the occurrence of spatial and spatiotemporal clusters in São Paulo municipalities and micro-regions was analyzed using geoprocessing techniques.

## METHODS

This is a population-based ecological study, using secondary data from the Brazilian Notifiable Diseases Information System (SINAN – *Sistema de Informação de Agravos de Notificação* ) for meningitis, in the state of São Paulo, from January 2005 to December 2013. SINAN is a nationwide information system, free for use in the public domain, aimed at recording and processing data on mandatory reporting diseases in all levels of health care services, in both public and private sectors. Data were retrieved from SINAN database using the 10^th^ revision of the International Classification of Diseases (CID-10) code for pneumococcal meningitis (PM – G00.1)^6^ .

Data on PCV10 coverage in São Paulo from 2010 to 2013 were collected from the Department of Informatics of the Brazilian Unified Health System (DATASUS)^7^ . Vaccine coverage was defined as the number of third PCV10 doses administered to children aged < 1 year, divided by the estimated target population in a given area and year, multiplied by 100.

The 2010 São Paulo Index of Social Responsibility (IPRS – *Índice Paulista de Responsabilidade Social* ) was used as a socioeconomic variable in analyzing the 63 administrative micro-regions and the 645 municipalities of São Paulo^8^ . The IPRS is a composed index that, in addition to the HDI dimensions (income *per capita* , longevity, and education level), includes updated administrative information to detect changes in the living conditions in periods shorter than the 10 years that separate the Brazilian demographic censuses, the source of information composing the municipal HDI. The IPRS allows for detailing the living conditions in São Paulo cities, a cornerstone for planning public policies. The IPRS is divided into five groups, in which five is the group with the lowest wealth and the worst social indicators and one is the group with the highest wealth and the best social indicators^8^ .

For analyzing the 63 São Paulo micro-regions, data from the municipalities were grouped according to the same criteria and dimensions of the municipal IPRS.

### Descriptive Analysis

The PM incidence rates in under-5 children according to São Paulo municipalities and micro-regions were calculated based on SINAN data and the resident population estimated by the Brazilian Institute of Geography and Statistics (IBGE)^9^ . The 2000 census data was used for the period from 2005 to 2009 and the 2010 census data, for the 2010–2013 period^10^ . Due to the lack of data on the population distribution by municipalities in 2013, the geographic distribution and spatial analysis in the post-vaccination period was restricted to 2011–2012. Data from the Information System on Live Birth (SINASC – *Sistema de Informação de Nascidos Vivos* ) was used to calculate the PM incidence rate in children < 1 year old, in 2013^7^ .

### Spatial Analyses

Spatial analysis used a Geographic Information System (GIS) (SIRGAS 2000 datum) for spatial data representation, in which the estimated PM incidence rates in under-5 children, vaccine coverage, and socioeconomic variables were associated with the spatial data.

The PM incidence rates were analyzed by São Paulo municipalities and micro-regions. These data, together with data on vaccination coverage and socioeconomic variables, were used to prepare the thematic maps with QGIS 2.6.1 software, using the IBGE cartographic base^11 , 12^ .

To detect spatial and spatiotemporal clusters of PM cases, São Paulo municipalities were used as ecological units of analysis through the scan statistic technique, using SaTScan 9.2^TM^, and the significance of the clusters found was evaluated through the likelihood ratio test, obtained through Monte Carlo simulation^13 , 14^ .

Relative risk (RR) allows one to compare information from different areas, unifying and removing the effect of distinct populations, showing the intensity of the occurrence of a given phenomenon regarding all other regions studied, being considered significant at p < 0.05.

The number of PM cases per São Paulo micro-region was modeled through a Besag-York-Mollié model, using a zero-inflated Poisson regression and considering minimally informative priors through software R version 3.3.2 with the integrated nested Laplace approximation (INLA) package^15^ .

Each IPRS dimension was considered a covariant in the analysis of PM incidence rates from 2005 to 2009 and from 2011 to 2012. The analysis unit was each São Paulo micro-region. To investigate the relationship between PM occurrence and the covariates of interest, a Bayesian inference for latent Gaussian model was used, using a zero-inflated Poisson model due to the excess of analyzed units with zero cases. The INLA methodology, which is an alternative to the Markov chain Monte Carlo, was used in the model since this is computationally more efficient for the type of modeling used in this study^15^ .

## RESULTS

**Figure 1:**
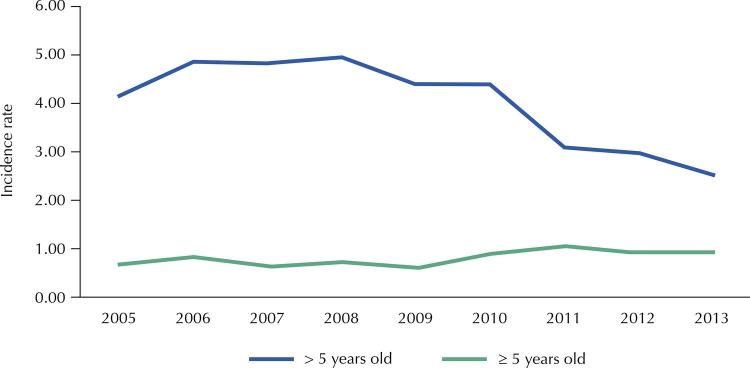
Pneumococcal meningitis incidence rate (per 100,000 inhabitants), by age group and year of occurrence. São Paulo, Brazil, 2005–2013.

From January 2005 to December 2013, 3,963 PM cases were reported in São Paulo. Total incidence rates remained stable throughout the study period (1/100,000 inhabitants in 2005 to 1.06/100,000 in 2013), and the highest rates occurred among infants. In the post-vaccination period, there was a decrease in PM incidence rates in under-5 children, particularly in infants (from 13.1/100,000 in 2005 and 14.38/100,000 in 2009 to 8.69/100,000 in 2011). There was a trend towards higher PM incidence rates among adults over 50 years old, albeit to a lesser extent than the drop in incidence rates in children ( Table 1 and Figure 1 ).

**Table 1:** Pneumococcal meningitis incidence rates (per 100,000 inhabitants), by year of occurrence and age group. São Paulo, Brasil, 2005 to 2013a.

Age group	Year								

	2005	2006	2007	2008	2009	2010^b^	2011	2012	2013
< 1	13.11	16.11	15.30	14.50	14.38	13.13	8.69	8.45	8.51
1–4	1.99	2.11	2.29	2.60	1.92	2.24	1.64	1.54	0.98
5–9	0.75	1.17	0.98	0.44	0.83	1.01	0.94	0.93	0.60
10–14	0.40	0.62	0.42	0.30	0.54	0.57	0.75	0.74	0.65
15–19	0.38	0.58	0.35	0.47	0.27	0.58	0.78	0.45	0.41
20–29	0.48	0.61	0.33	0.44	0.38	0.66	0.64	0.32	0.69
30–39	0.54	0.78	0.66	0.61	0.57	0.77	0.82	0.80	0.70
40–49	0.80	0.83	0.61	0.91	0.84	1.12	1.37	1.20	1.38
50–59	1.40	1.27	1.04	1.69	0.82	1.58	1.70	1.74	1.49
60–69	1.13	1.60	1.30	1.09	0.82	1.32	1.76	1.68	1.42
70–79	1.50	1.26	0.91	0.88	1.14	0.75	1.43	1.22	1.31
> 80	1.21	0.56	1.07	0.84	0.64	1.35	1.19	1.33	1.62

Total	1.00	1.16	0.96	1.02	0.88	1.15	1.20	1.07	1.06

^a^ 65 cases without age group information.

^b^ Year of PCV10 introduction.

In the pre-vaccination period (2005–2009), most PM cases among infants occurred in those aged from six to 12 months. After vaccination, the proportion of cases in the second semester of life decreased, with most cases occurring in the first four months of life. In 2013, more than 40% of cases in infants occurred in those two to four months old ( Table 2 ).

**Table 2:** Distribution (%) of pneumococcal meningitis (PM) cases in infants, by age group and year of occurrence. São Paulo, 2005–2013.

Year/%	Group age (months)			

	< 2	2 ⱶ 4	4 ⱶ 6	6 ⱶ 12
2005	15.56	21.11	11.11	52.22
2006	16.30	18.48	28.26	36.96
2007	22.09	13.95	23.26	40.70
2008	6.25	26.25	15.00	52.50
2009	21.79	15.38	15.38	47.44
2010	24.29	20.00	24.29	31.43
2011	32.65	20.41	22.45	24.49
2012	31.25	31.25	12.50	25.00
2013	23.08	40.38	13.46	23.08

The distribution of annual PM incidence rates in under-5 children was 4.8/100,000 inhabitants-year in the pre-vaccination period and 2.9/100,000 inhabitants-year in the post-vaccination period, with a large variation among municipalities. In the city of São Paulo, annual PM incidence rates in under-5 children were 6.9 and 3.7/100,000 inhabitants-year in the pre- and post-vaccination periods, respectively.

**Figure 2:**
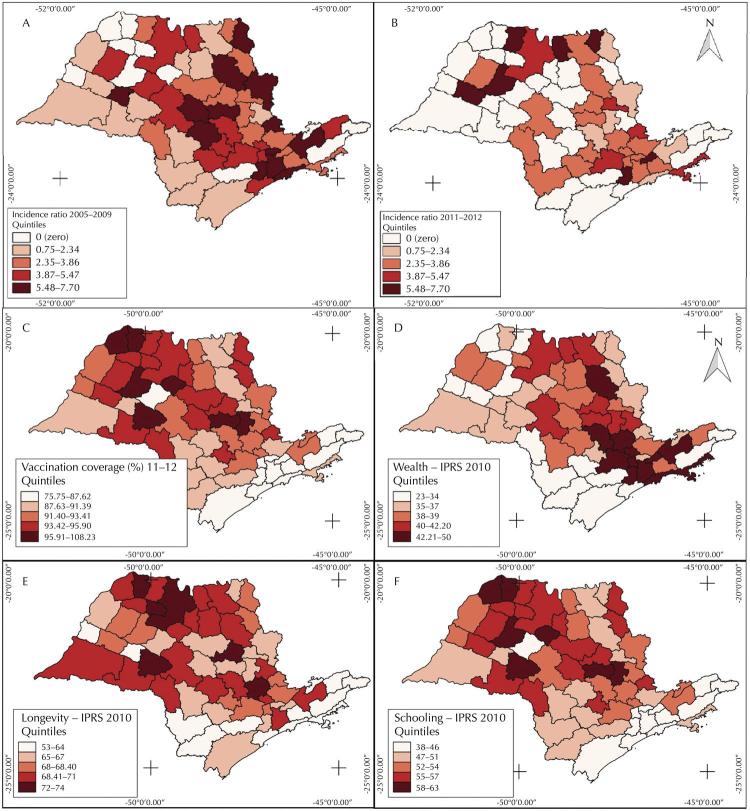
Distribution of pneumococcal meningitis (PM) incidence rates in under-5 children (per 100,000 inhabitants), before (A) and after (B) introduction of 10-valent pneumococcal vaccine (PCV10) in childhood immunization; coverage (%) of the third PCV10 dose from 2011 to 2012 (C); socioeconomic variables wealth 263 (D), longevity (E), and schooling (F) in quintiles in 2010, by São Paulo state micro-regions. IPRS: São Paulo Index of Social Responsibility

In all age groups, PM cases occurred in 264 out of 645 municipalities from 2005 to 2009, and in 185 from 2011 to 2013. Considering only cases in under-5 children, just 145 and 63 municipalities reported PM cases in the two periods, respectively. The large number of municipalities with zero cases impaired the analysis of PM incidence rates distribution. Therefore, the analysis was performed by micro-regions ( Figure 2 ) since aggregating data in larger geographic areas allows for a better visualization.

**Figure 3:**
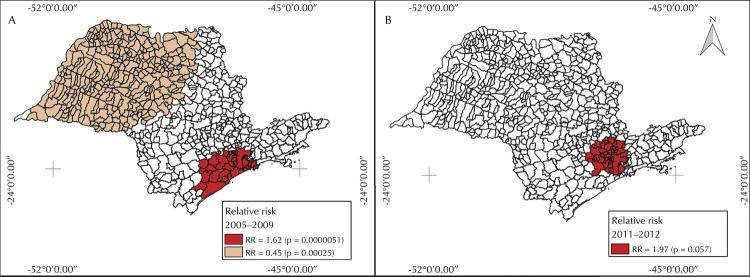
Spatial clusters of the mean annual pneumococcal meningitis (PM) incidence rates in under-5 children (per 100,000 inhabitants), by São Paulo state municipalities, in the pre- (2005–2009) (A) and post-vaccination (2011–2012) (B) periods.

Scan statistics was used to detect spatial clusters of municipalities with higher or lower RR and higher or lower PM incidence rate ( Figure 3 ). Two clusters were observed in the pre-vaccination period ( Figure 3 , A). The first one, with a RR = 1.62 (p = 0.0000) for PM incidence, included 39 municipalities in the southeast of the state (including São Paulo city, with 1,166,547 under-5 children and PM incidence rate of 6.5/100,000/year). The second cluster, with a RR = 0.45 (p = 0.0003) for PM incidence, included 306 municipalities in the northwest of the state, with a population of 336,865 under-5 children and PM incidence rate of 2.5/100,000/year.

Only one cluster, albeit not a significant one, of higher PM incidence rate (RR = 1.97 and p = 0.0570) was observed in the post-vaccination period, comprising 47 municipalities (including São Paulo city) with a population of 1,310,494 under-5 children and PM incidence rate of 4.1/100,000/year ( Figure 3 , B).

The relationship between PM incidence rates in under-5 children and the covariates of wealth, longevity, education level and vaccination coverage were analyzed for each micro-region ( Table 3 ). The odds of zero-PM cases were 0.517 in the pre-vaccination period and 0.601 in the post-vaccination period. Prior to vaccination, the IPRS wealth variable was positively associated with PM occurrence (RR = 1,026; 95%CI 1,002–1,052), whereas in the post-vaccination period none of the IPRS covariates were associated with PM occurrence.

**Table 3:** Bayesian Besag-York-Mollié model (zero-inflated Poisson) for pneumococcal meningitis incidence rates in under-5 children and relationship with the covariates wealth, longevity, schooling and vaccination coverage, in the pre- (2005–2009) and post-vaccination (2011–2012) periods, by São Paulo state micro-regions.

Variable	Covariables model					

	2005–2009			2011–2012		
	
	Mean*	Qt 0.025	Qt 0.975	Mean*	Qt 0.025	Qt 0.975
p (0)	0.517	0.462	0.572	0.601	0.515	0.684
Intercept	0.647	0.112	3.561	0.249	0.008	6.718
Wealth	1.026	1.002	1.052	1.031	0.988	1.078
Longevity	0.995	0.963	1.030	1.008	0.953	1.072
Schooling	0.992	0.968	1.016	0.986	0.952	1.020
Vaccination coverage	-	-	-	1.001	0.995	1.007

* Except for p = 0, which is the probability of zero, the mean represents the relative risk.

Qt: 95% credibility interval.

## DISCUSSION

In São Paulo, during the entire studied period (2005–2013), the highest PM incidence rates occurred in under-5 children, mainly in infants. A reduction in PM incidence rates was observed in the post-vaccination period. Thematic maps showed higher PM incidence rates among under-5 children in São Paulo micro-regions with higher wealth index in the pre-vaccination period. This effect disappeared in the post-vaccination period. In the pre-vaccination period, two clusters were found, one of low PM risk in the northwest of the state, and one of high risk in the southeast region, which includes São Paulo city. Only the latter cluster persisted in the post-vaccination period. In the Bayesian model analysis, only wealth was positively associated to PM in before the vaccination period.

The observed reduction in PM incidence rates in under-5 children, two years after the PCV10 introduction, is similar to results of other Brazilian studies, as well as those observed in other countries after pneumococcal conjugate vaccines introduction^18^ . The reduction from 56% to 69% in IPD incidence rates, including meningitis, in children younger than two years of age was observed after 7-valent pneumococcal conjugate vaccine (PCV7) introduction in the United States and Australia^23 , 24^ .

In São Paulo, from 2010 to 2013, PCV10 primary vaccination was administered to children at three, five, and seven months of age. After the vaccination program implementation, the greatest reduction in PM incidence rates occurred among infants aged from six to twelve months, who must have already completed the primary vaccination schedule. In the post-vaccination period, more than 40% of PM cases in infants occurred in those aged from two to four months. In 2014, in face of these changes in PM pattern, São Paulo has modified the vaccination schedule and started administering PCV10 at two, four and six months of age, like the rest of the country. In 2016, a 2+1 schedule was adopted in Brazil^25^ . A Brazilian case-control study showed an effectiveness of 81.3% for one PCV10 dose and of 95.5% for three PCV10 doses in protecting against IPD caused by the vaccine serotypes^26^ .

Our results did not show a decrease in PM incidence rates in older age groups that are not target for vaccination, corroborating the findings of other studies. Two retrospective studies using hospital data failed to detect a significant effect on IPD incidence in unvaccinated populations two years after PCV10 introduction^27 , 28^ . No decrease on IPD was observed in unvaccinated individuals from 2 to 17 years old, whereas an increase was observed in adults aged ≥ 18 years, in an interrupted time series analysis using data from the National Reference Laboratory (all IPD cases) and SINAN (only PM cases), adjusted for seasonality and temporal trends, in the pre- (2008–2009) and post-vaccination (2011–2013) periods^19^ . A review of the impact of PCV10 in Brazil found inconsistent data regarding herd protection^29^ .

After pneumococcal conjugate vaccines introduction in routine childhood immunization programs, the decline on overall IPD in under-5 children is substantial and consistent amongst different studies. Reduction of IPD rates in older age groups that are not target of vaccination (indirect effects) has been more variable and subject of debate^30 , 31^ . The indirect effects of PCV childhood immunization may vary from place to place according to disease epidemiology before vaccine introduction (incidence rates, serotype distribution), vaccination program characteristics (which vaccine is used, number of doses in the primary schedule, vaccine coverage, catch-up implementation), and the degree of pneumococcal serotypes replacement. Longer observation time may be required to detect the indirect effects of vaccination^32^ . A meta-analysis of the impact of pneumococcal vaccination programs found that seven to 10 years may be necessary to achieve substantial herd protection^31^ . In addition, in places with a low burden of disease, adult pneumococcal colonization is rare and the childhood vaccination program has a major impact on pneumococcal transmission in the community. On the other hand, in places with a higher burden of disease, adults are more frequently colonized and may have greater role in transmission and, consequently, the indirect effects of childhood vaccination program may be more limited^33^ .

In our study, the PM incidence rate was positively associated to wealth, which was unexpected. Data from different countries showed that crowded living conditions, less education, and lower socioeconomic status are risk factors for pneumococcal disease^1^ .

Although blood and cerebral spinal fluid cultures are considered the standard for PM diagnosis, their sensitivity is limited. Prior antibiotic use and improper clinical specimens’ handling or transport may jeopardize culture results. More sensitive tests, based on molecular techniques and pneumococcal antigens detection, are available^34^ . In São Paulo, the first state to introduce PCR in public health laboratories, the proportion of bacterial meningitis of unknown etiology decreased from approximately 50% in the beginning of the 2000s to about 25% in 2010–2013. The incorporation of real-time PCR into routine microbiological methods increased pneumococcal detection by 52%^37^ . The use of immune-chromatography test (ICT) for pneumococcal antigens in meningitis diagnoses also increases pneumococcal detection in blood and cerebral spinal fluid, particularly in the case of previous antibiotic use^36^ . However, the use of PCR and ICT for pneumococcal antigen is limited in clinical practice. In Brazil, although PCR is already available in central public health laboratories (LACEN) in some Brazilian states such as São Paulo, its use in clinical practice is still limited to more developed regions, larger laboratories, and some university hospitals.

As described above, rates of bacterial meningitis cases with a definitive etiological diagnosis have increased progressively from 36.4% in 2005 to 53.2% in 2013, mainly due to PCR incorporation in meningitis diagnosis; however, the current rates are still low. Even the cytological examination of blood and cerebral spinal fluid was performed in only 65.4% of cases in 2013, without an increase in this rate in recent years^38^ . Additionally, medical practice regarding blood cultures collection also varies in different regions and health services.

SINAN, the data source for this study, is a passive system, dependent on technical operation conditions of the epidemiological and laboratory surveillance system of each geographical area to detect, notify, investigate, and perform specific laboratory tests for the etiological diagnosis of bacterial meningitis. Underreporting is an issue. A meningitis study conducted in Belo Horizonte, in the state of Minas Gerais, in Southeastern Brazil, showed that SINAN data was not complete, with an estimated sensitivity of 66% for all-causes meningitis^39^ . This is the main limitation of our data.

Consequently, PM is probably underdiagnosed and underreported in Brazil. Brazil is a huge country with great regional socioeconomic differences. The State of São Paulo also has regional differences, with areas of lower socioeconomic development. Our hypothesis is that the proper PM diagnosis and reporting is not equally distributed in different regions – wealthy areas with better access to health care, better laboratory structure, greater use of more sensitive diagnostic tests and better surveillance have greater proportion of reported cases of meningitis with definitive etiological diagnosis. Furthermore, the proportion of meningitis cases with unknown etiology may be greater in less developed areas. These regional differences might explain the association found between PM rates and wealth.

The coverage of the third PCV10 dose increased quickly after vaccine introduction in Brazil, from 24% in 2010 to 93% in 2013. However, vaccine coverage rates varied by municipality, as showed in our thematic maps. Coverage rates were satisfactory and homogeneous, with all micro-regions presenting vaccine coverage > 75%. This could explain why vaccination coverage was not important in the Bayesian model analysis for the PM incidence rates in under-5 children by São Paulo micro-regions.

Surveillance data used in this study did not identify pneumococcal serotypes; thus, we could not evaluate the impact of vaccination on the PM cases caused by vaccine and non-vaccine serotypes. Despite the limitations, there are many reasons for using ecological studies in epidemiology, among which we highlight the low cost of working with secondary data and the interest in ecological effects^40^ .

Health scenarios designed by geoprocessing techniques add socioeconomic, environmental and structural health data to the geographic component. The spatial correlation of data allows for planning strategic interventions in a given geographic area, through the characterization of regional scenarios^41^ . The spatial statistical analysis carried out in this study contributes to the evaluation of PM cases and clusters in São Paulo. We highlight the methodology used to evaluate the associations between PM and socioeconomic variables. Although the Poisson model is used in studies to map diseases, it may be inappropriate in some applications due to the excess of zeros in the data compared to what is expected for the model – a situation found in this study. To deal with this scenario, we employ a zero-inflated model that allowed modeling with consistent results^42^ . Modeling spatiotemporal epidemiological data with excess zeros requires Bayesian methods, which generally demand a number of inferential processes, generating excessive computational time. In this study, we highlight the use of the INLA method proposed by Rue et al.^15^ This method allowed us to analyze PM cases considering the above conditions with an expressive computational gain, enabling several models to be adjusted and several scenarios to be explored to solve this problem, which would not be possible in models that did not simultaneously incorporate zero counts and spatiotemporal structure^42^ .

Results of this study emphasize the need to invest in the meningitis diagnostic capacity in Brazil, to improve the quality of disease surveillance and recording and to optimize vaccine coverage in regions with low coverage.

Further studies are required to monitor pneumococcal disease epidemiology, the effects of PCV10 introduction, and the pneumococcal serotypes distribution in the Brazilian population. Studying smaller ecological units would also reduce the effect of relative over-dispersion and facilitate interventions in geographically smaller areas and in those areas of higher risk.
